# Supplementary material for: CXCR4 mediated recognition of HIV envelope spike and inhibition by CXCL12
Source: Nat Commun. 2025 Sep 30;16:8653. doi: 10.1038/s41467-025-63815-2 (PMC12485088; doi:10.1038/s41467-025-63815-2)
Supplement: Supplementary file 1 — Supplementary Information [file 41467_2025_63815_MOESM1_ESM.pdf]

Zhang et al (2025)

Supplementary Information for:

CXCR4 chemokine receptor: HIV envelope spike recognition and cognate CXCL12 inhibition

Zhiying Zhang, Hongwei Zhang, Lyuqin Zheng, Shihua Chen,
Shuo Du, Junyu Xiao and Dinshaw J. Patel

This pdf contains:

- Supplementary Figures 1-12
- Supplementary Table 1

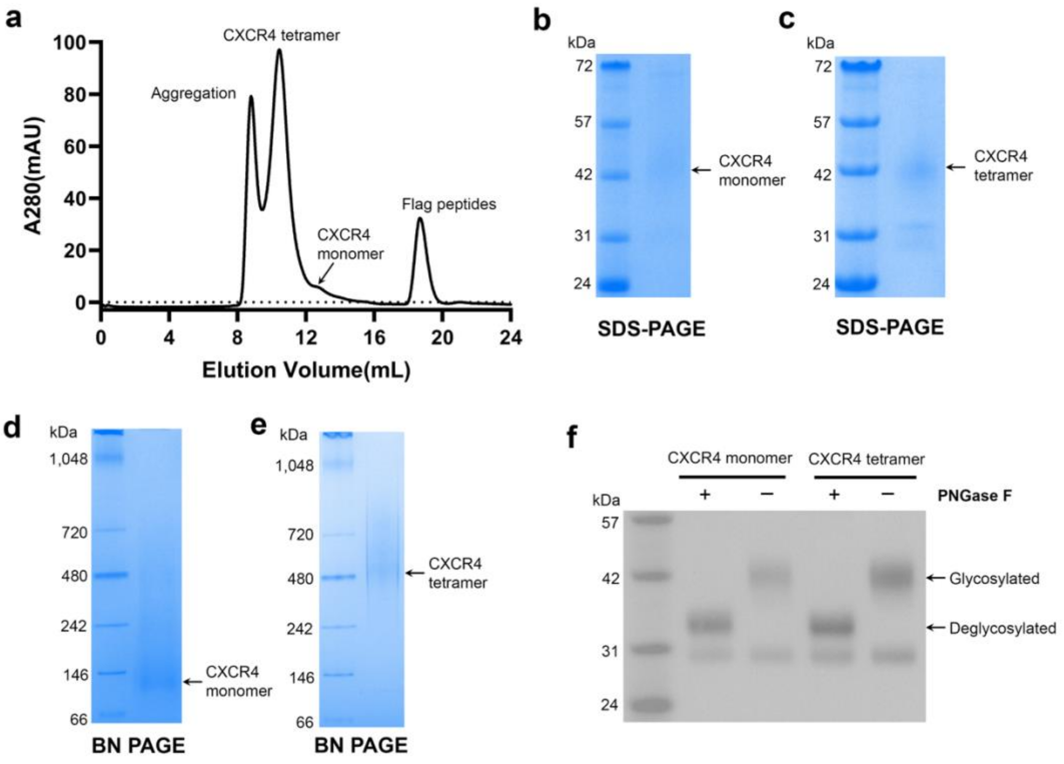

Supplementary Figure 1. **Protein purification and biophysical characterization of CXCR4. a** Size exclusion chromatography (SEC) of purified CXCR4 expressed in mammalian cells showing distinct peaks corresponding to aggregates, tetramers, monomers, and free Flag peptides. **b,c** SDS-PAGE analysis confirms the monomeric (b) and tetrameric (c) CXCR4 bands related to a. **d,e** Blue native PAGE (BN-PAGE) analysis showing distinct bands for CXCR4 monomer (d) and tetramer (e). **f** Western blot analysis with and without PNGase F treatment confirms glycosylation of CXCR4 in both monomeric and tetrameric forms.

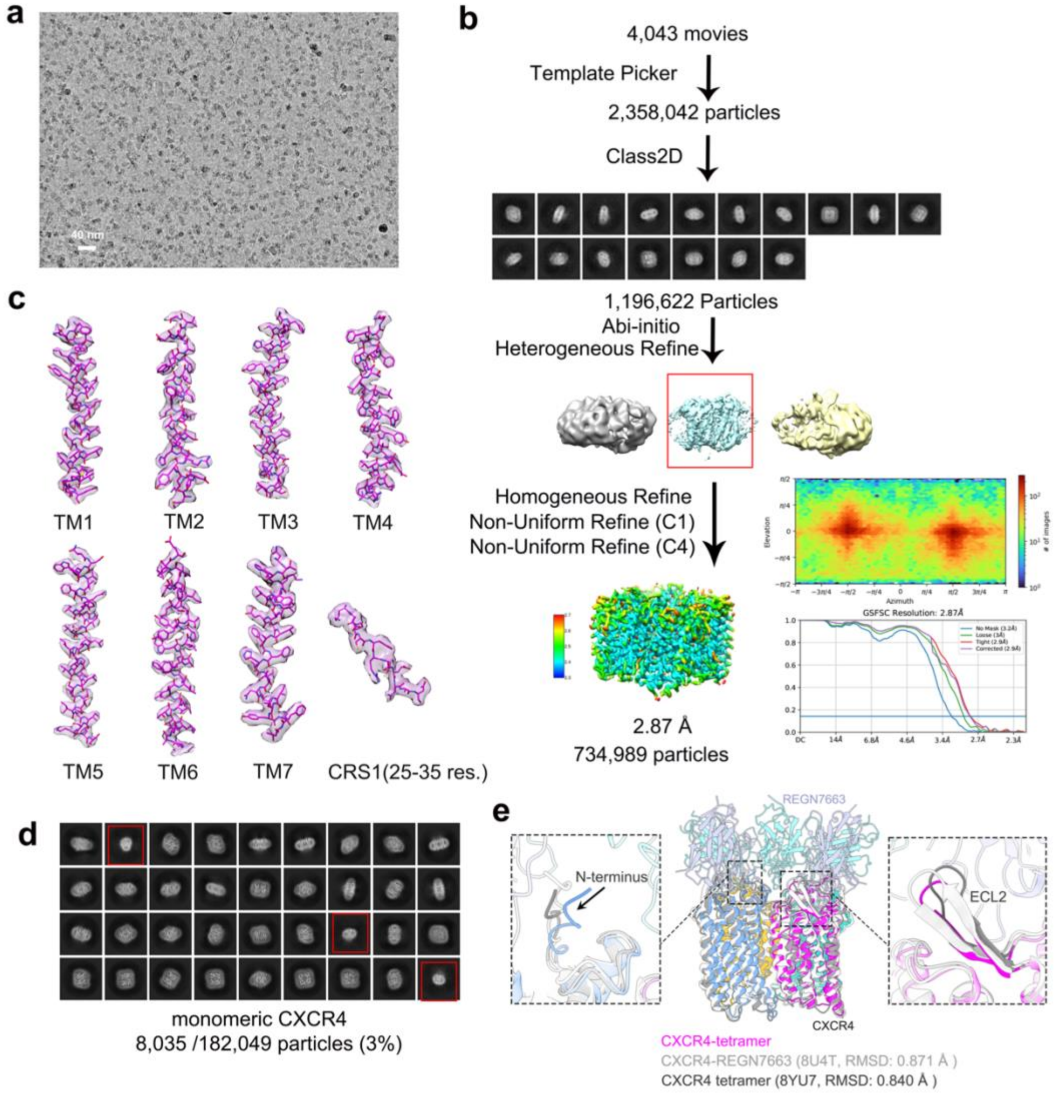

Supplementary Figure 2. **Image processing of CXCR4 tetramer. a** A representative raw micrograph of CXCR4 tetramer. **b** Image processing workflow, including a map colored by local resolution, a local resolution estimate based on the FSC 0.143 cutoff, and a representation of the angular distribution of particles used in the final reconstruction. **c** Local density mapping of TM1-7 and CRS1. **d** Class averages showing a minor population of monomeric CXCR4 particles. From 182,049 total particles in this subset, 8,035 particles (~3%) were classified as monomeric (highlighted in red boxes). **e** Structural alignment of the CXCR4 tetramer (magenta) with the CXCR4–REGN7663 complex (PDB: 8U4T, light gray) and CXCR4 tetramer (PDB: 8YU7, dark gray), showing high overall similarity (RMSD = 0.871 Å and 0.840 Å, respectively). The central panel displays the aligned structures, with Fab fragments (REGN7663) shown in light blue and purple. Zoomed-in views highlight minor structural differences at the N-terminus (left panel) and ECL2 loop (right panel), which are involved in Fab binding.

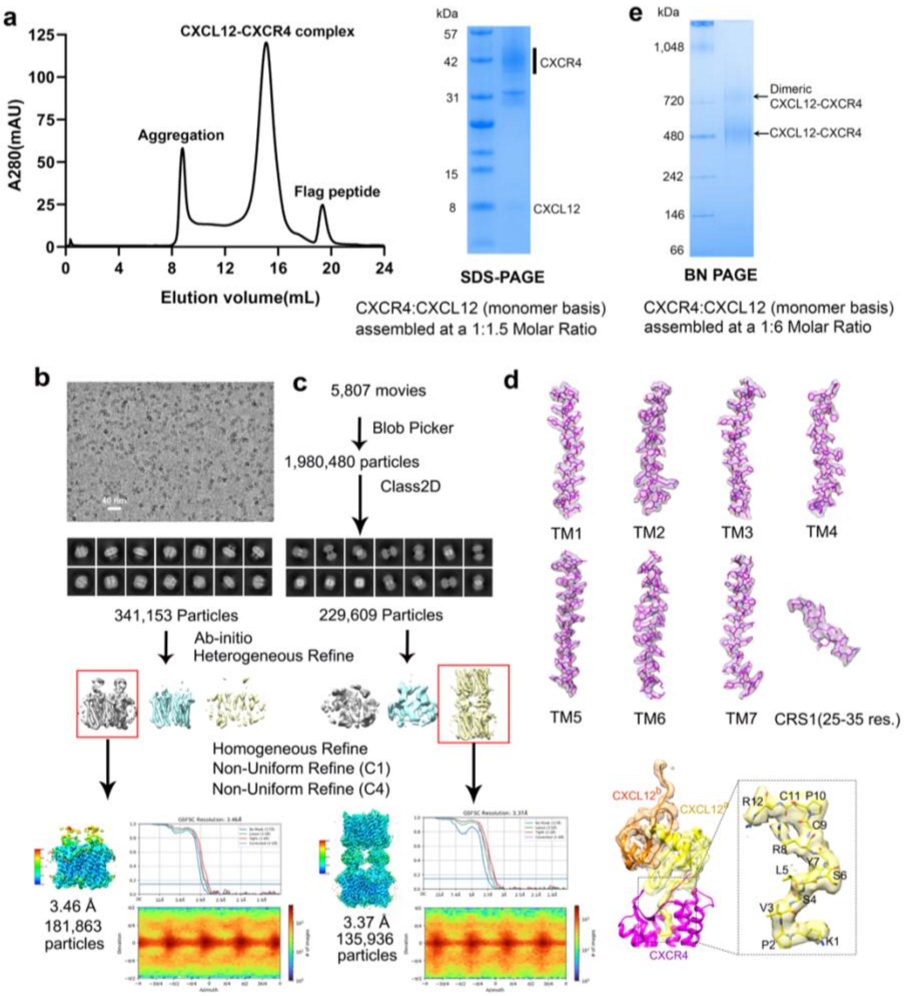

Supplementary Figure 3. **Protein purification and Image processing of CXCL12-CXCR4 complex. a** Size-exclusion chromatography (SEC) and SDS-PAGE confirm successful co-expression of the CXCR4-CXCL12 complex at a 1:1.5 molar ratio (monomer basis). **b** A representative raw micrograph of CXCL12-CXCR4 complex, including a map colored by local resolution, a local resolution estimate based on the FSC 0.143 cutoff, and a representation of the angular distribution of particles used in the final reconstruction. **c** Image processing workflow. **d** Local density mapping of TM1-7, CRS1 and N-terminus of CXCL12 (Residues K1 to R12). Amino acids are labeled in boxed panel. **e** BN-PAGE analysis of CXCL12-CXCR4 complexes reveals the formation of both 8:4 and 8:8 stoichiometries when assembled at a 1:6 molar ratio (monomer basis).

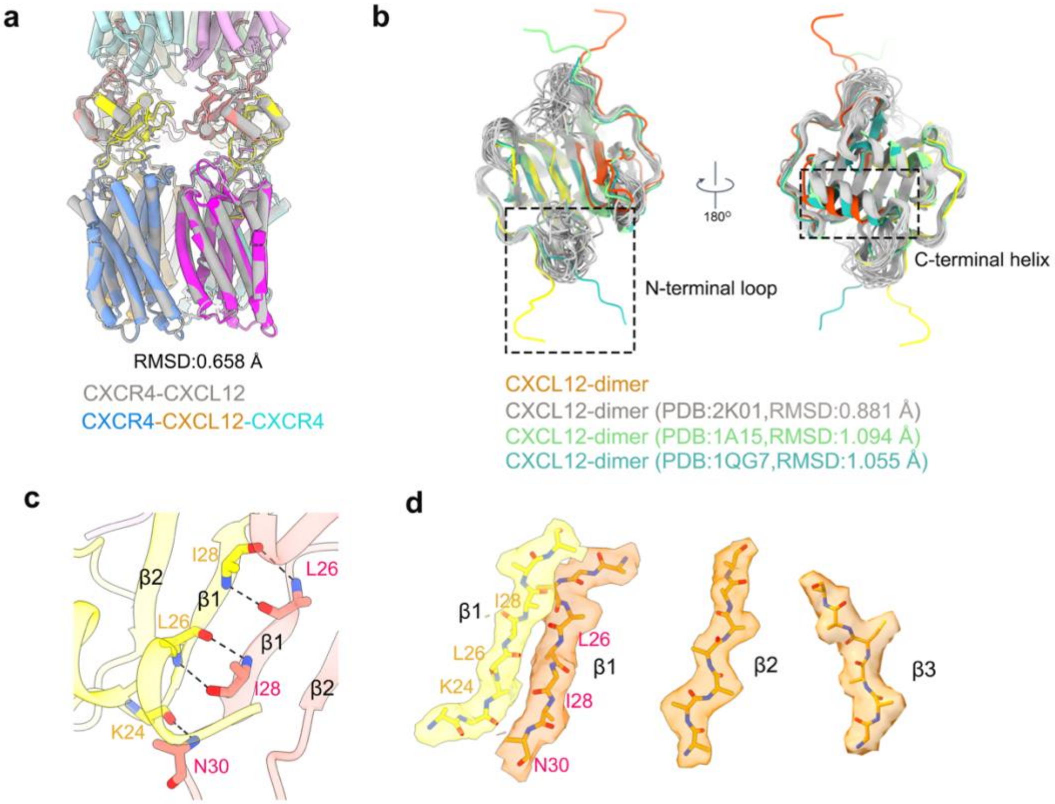

Supplementary Figure 4. **Structural alignment of two conformations of CXCL12-CXCR4 complex and CXCL12 dimer with its crystal structure. a** Comparison of the two conformations of the CXCL12-CXCR4 complex. **b** Comparison of the CXCL12 dimer in our structure (in color yellow and orange) with those in PDB entries 1QG7 (in dark green), 1A15 (in light green), and 2K01 (in sliver), which represent the mammalian crystal structure, a synthetic structure, and an NMR structure, respectively, showing notable differences at the N-terminal loop and C-terminal helix. **c** CXCL12 dimer interface, with labeling of interfacial residues. **d** Local density of the CXCL12 dimer interface, emphasizing β1. The local density of β2 and β3 is also presented.

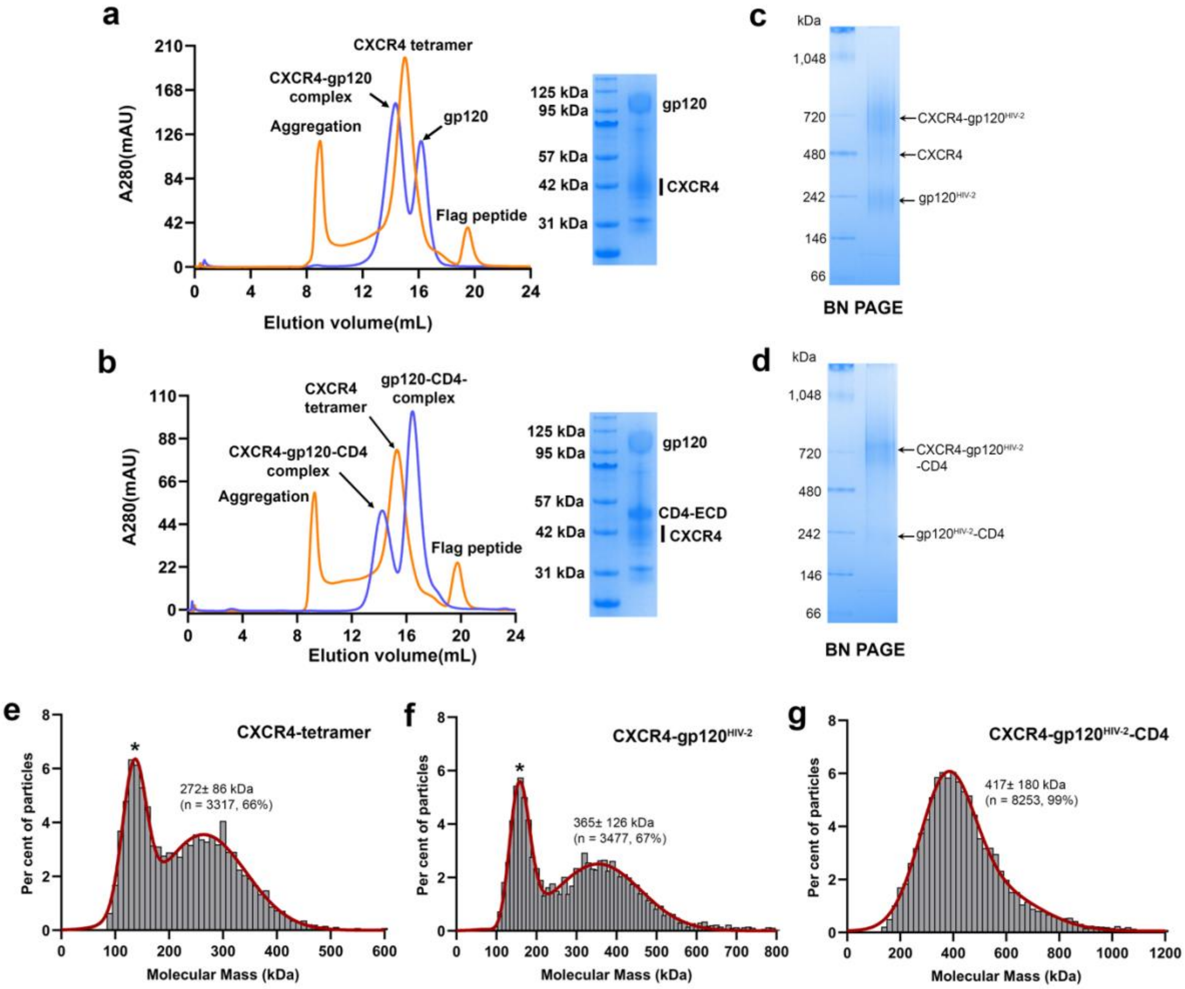

Supplementary Figure 5. **Protein purification and biophysical of CXCR4-gp120^{HIV-2} and CXCR4-gp120^{HIV-2}-CD4 complex. a** Size exclusion chromatography (SEC) and SDS-PAGE results for the CXCR4-gp120^{HIV-2} complex indicate a shift towards the CXCR4 peak. **b** SEC and SDS-PAGE of the CXCR4-gp120^{HIV-2}-CD4 complex demonstrate a significant shift towards the peaks corresponding to either CXCR4 or gp120^{HIV-2}-CD4. **c** Blue native PAGE (BN-PAGE) analysis reveals the formation of CXCR4-gp120^{HIV-2} complex. **d** BN-PAGE analysis of CXCR4 complexes with gp120^{HIV-2}-CD4 reveals the formation of distinct complexes. **e**-**g** Mass photometry analyses of ligand-free or ligand-bound complexes reveal molecular masses corresponding to CXCR4 tetramer (~272 kDa, e), CXCR4-gp120^{HIV-2} (~365 kDa, f), and the ternary CXCR4-gp120^{HIV-2}-CD4 complex (~417 kDa, g), with buffer blank signials highlighted by asterisks.

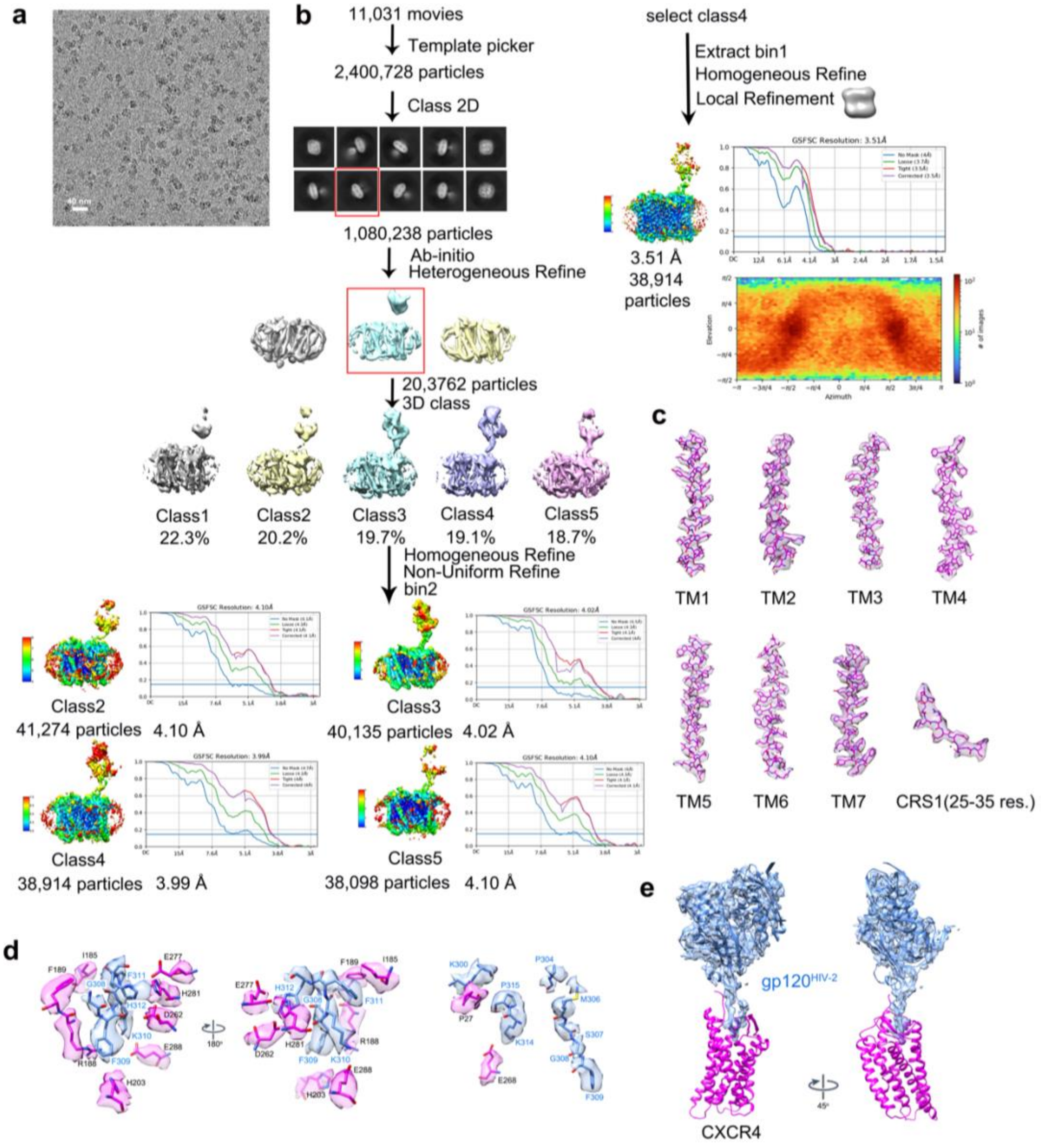

Supplementary Figure 6. **Image processing of CXCR4-gp120^{HIV-2} complex. a** A representative raw micrograph of CXCR4-gp120^{HIV-2} complex. **b** Image processing workflow, including a map colored by local resolution, a local resolution estimate based on the FSC 0.143 cutoff, and a representation of the angular distribution of particles used in the final reconstruction. **c** Local density mapping of TM1-7 and CRS1. **d** Representative local density maps of CXCR4-gp120^{HIV-2} interface. **e** Overview density map of gp120^{HIV-2}.

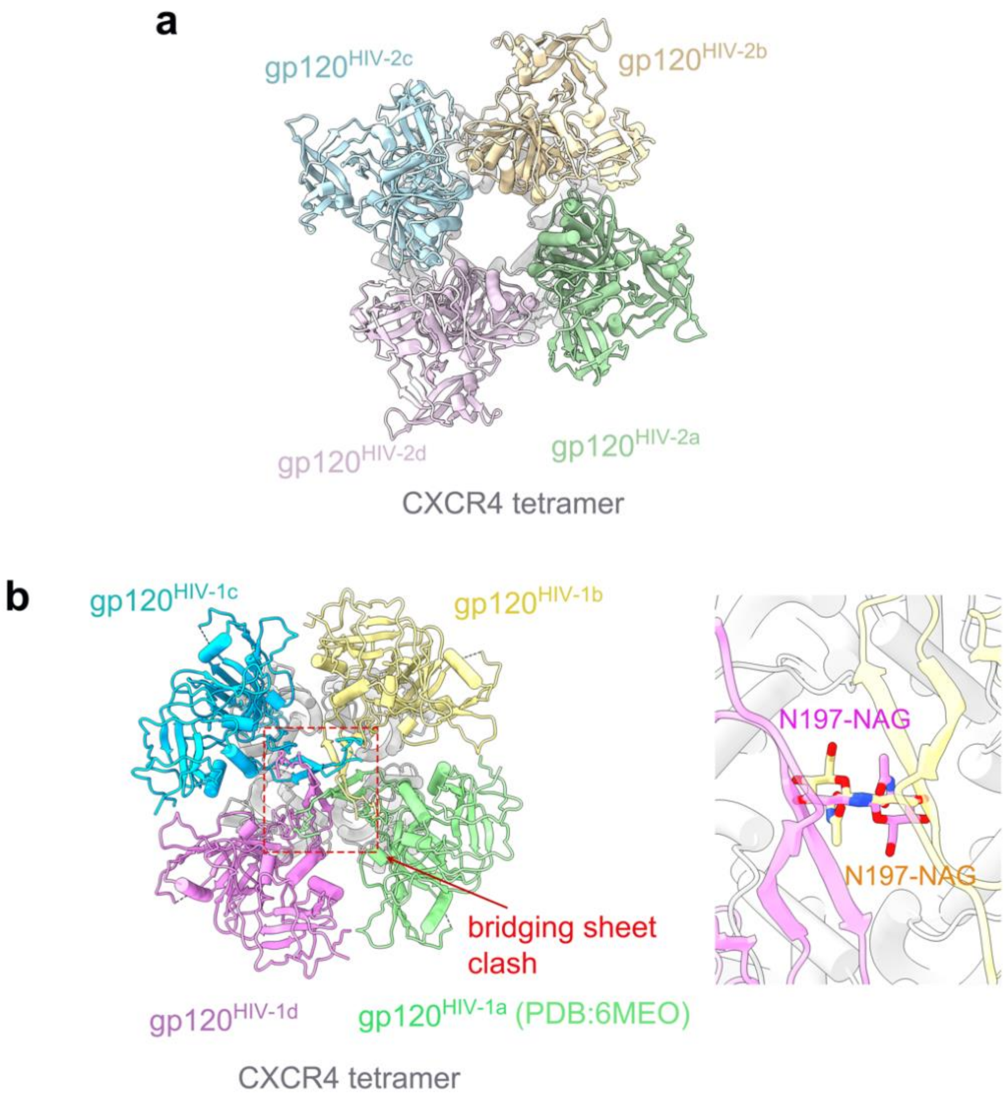

Supplementary Figure 7. **Structural modeling of tetrameric CXCR4 with four copies of HIV-1 or HIV-2 gp120. a** Structural model of tetrameric CXCR4 containing four copies of HIV-2 gp120. **b** Structural model of tetrameric CXCR4 containing four copies of HIV-1 gp120, with steric clash positions indicated in blow up segment.

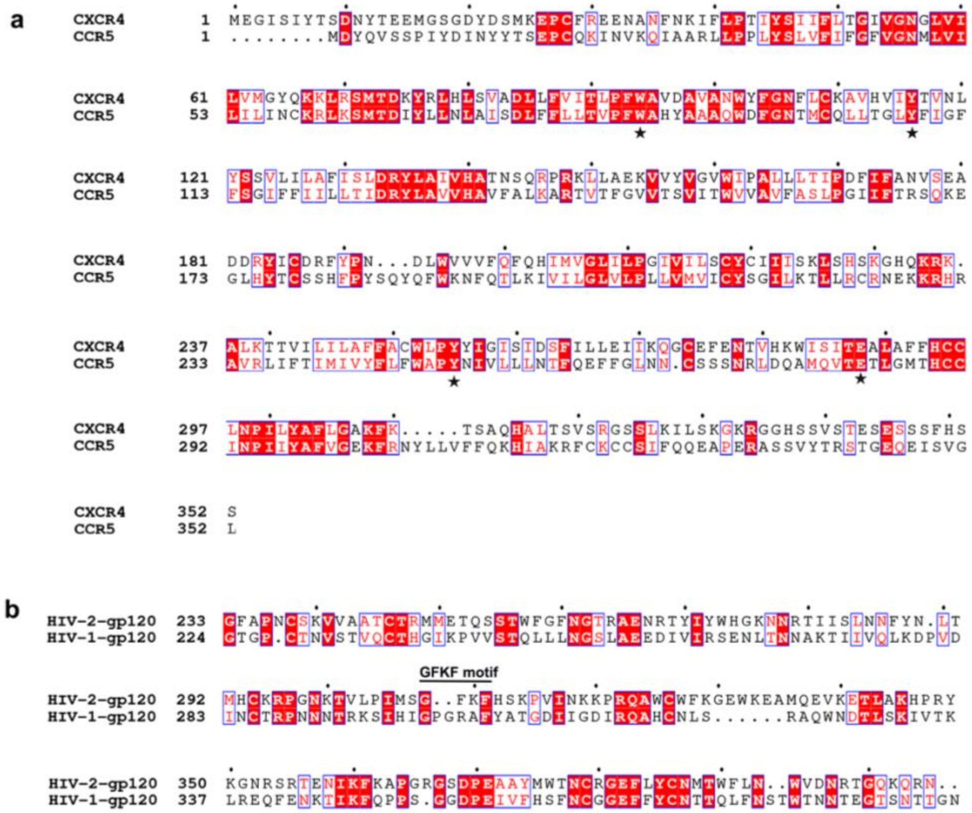

c

HIV2-gp120 (UniProt ref.:B2LT42)

MYRMQLLSCIALSLALVTNSKQFVTVFYGIPAWRNASIPLFCATKNRDTWGTIQCLPDNDDYQEIALNVTEAFDAWNNTV
TEQAVEDVWNLFETSIKPCVKLTPLCVAMNCTRNMTTSTGTTDTQNITIINDTSPCVRADNCTGLKEEEMVDCQFNMTGL
ERDKRKQYTEAWYSKDVICDNNTSSRSKCYMNHCNTSVITESCDKHYWDAMRFRYCAPPGFALLRCNDTNYSGFAPN
CSKVVAATCTRMMETQSSTWFGFNGTRAENRTYIYWHGKNNRTIISLNNFYNLTMHCKRPGNKTVLPIMSGFKFHSKPV
INKKPRQAWCWFKGEWKEAMQEVKETLAKHPRYKGNRSRTENIKFKAPGRGSDPEAAYMWTNCRGEFLYCNMTWFL
NWVDNRTGQKQRNYAPCHIRQIINTWHRVGKNVYLPPREGELTCNSTVTSIIANIDTGDQTDITFSAEVAELYRLELGDYK
LVEITPIGFAPTSVKRYSSAHQRHTRHHHHHH*

IL2-signal peptide – $gp120^{HIV-2}$(vcp strain, residues 23-501) – 6×His

d

HIV1-gp120 (UniProt ref.:Q70145)

MYRMQLLSCIALSLALVTNSNLWVTVYYGVPVWKEATTTLFCASDAKAYKAEVHNVWATHACVPTDPNPQEIVLENVTE
NFNMWKNNMVEQMHEDIISLWDQSLKPCVKLTPLCVTLNCIDLNNSTNNNNSSGVKTGIDKGEIKNCSFNTTTSVKDKEK
KEYALFYNLDVVQIGNDNTSYRLTSCNTSVITQACPKVTFEPIPIHYCTPAGYAILKCNGKKFNGTGPCTNVSTVQCTHGI
KPVVSTQLLLNGSLAEEDIVIRSENLTNNAKTIIVQLKDPVDINCTRPNNNTRKSIHIGPGRAFYATGDIIGDIRQAHCNLSRA
QWNDTLSKIVTKLREQFENKTIKFQPPSGGDPEIVFHSFNCGGEFFYCNTTQLFNSTWTNNTEGTSNTTGNDTITLPCRI
KQIVNMWQEVGKAMYAPPIKGKIKCSSNITGLLLTRDGGNNEMNTTEIFRPGGGDMRDNWRSELYKYKVVRIEPLGIAPT
RAKRRVVQREKRHHHHHH*

IL2-signal peptide – $gp120^{HIV-1}$(isolate 92BR020, residues 30-505) – 6×His

Supplementary Figure 8. **Sequence alignment. a** Sequence alignment of CXCR4 and CCR5, highlighting conserved residues with a black star below. **b** Alignment of the V3 loop sequences from HIV-1 and HIV-2, the GFKF motif of HIV-2 is indicated in the line. **c** The HIV-2 gp120 construct from the VCP strain, comprising residues 23–501, was used in this study (UniProt: B2LT42). **d** The HIV-1 gp120 construct from the 92BR020 strain, comprising residues 30–505, was used in this study (UniProt: Q70145).

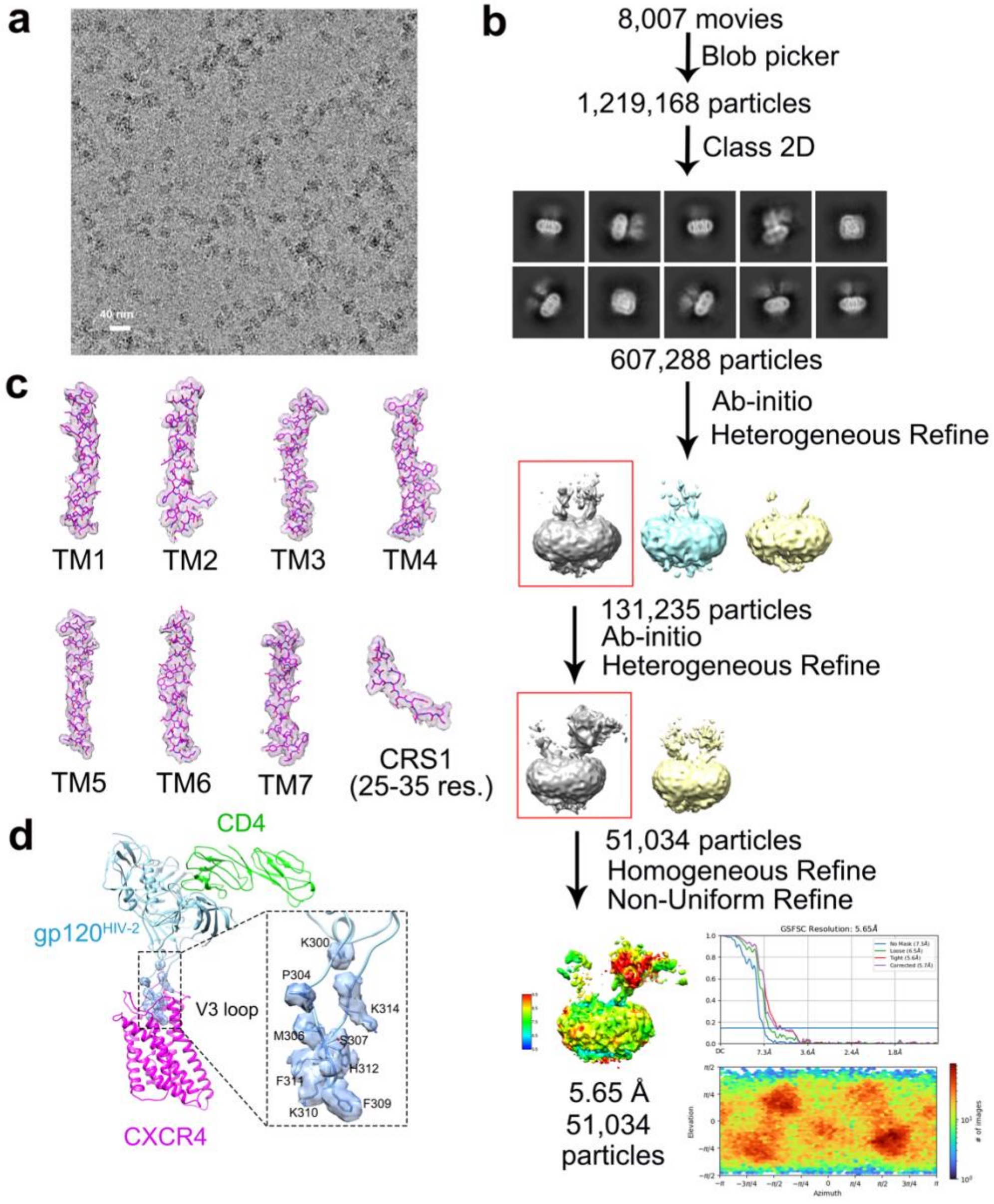

Supplementary Figure 9. **Image processing of CXCR4-gp120^{HIV-2}-CD4 complex**. **a** A representative raw micrograph of CXCR4-gp120^{HIV-2}-CD4 complex. **b** Image processing workflow, including a map colored by local resolution, a local resolution estimate based on the FSC 0.143 cutoff, and a representation of the angular distribution of particles used in the final reconstruction. **c** Local density mapping of TM1-7 and CRS1. **d** Representative local density maps of the V3 loop of gp120^{HIV-2}.

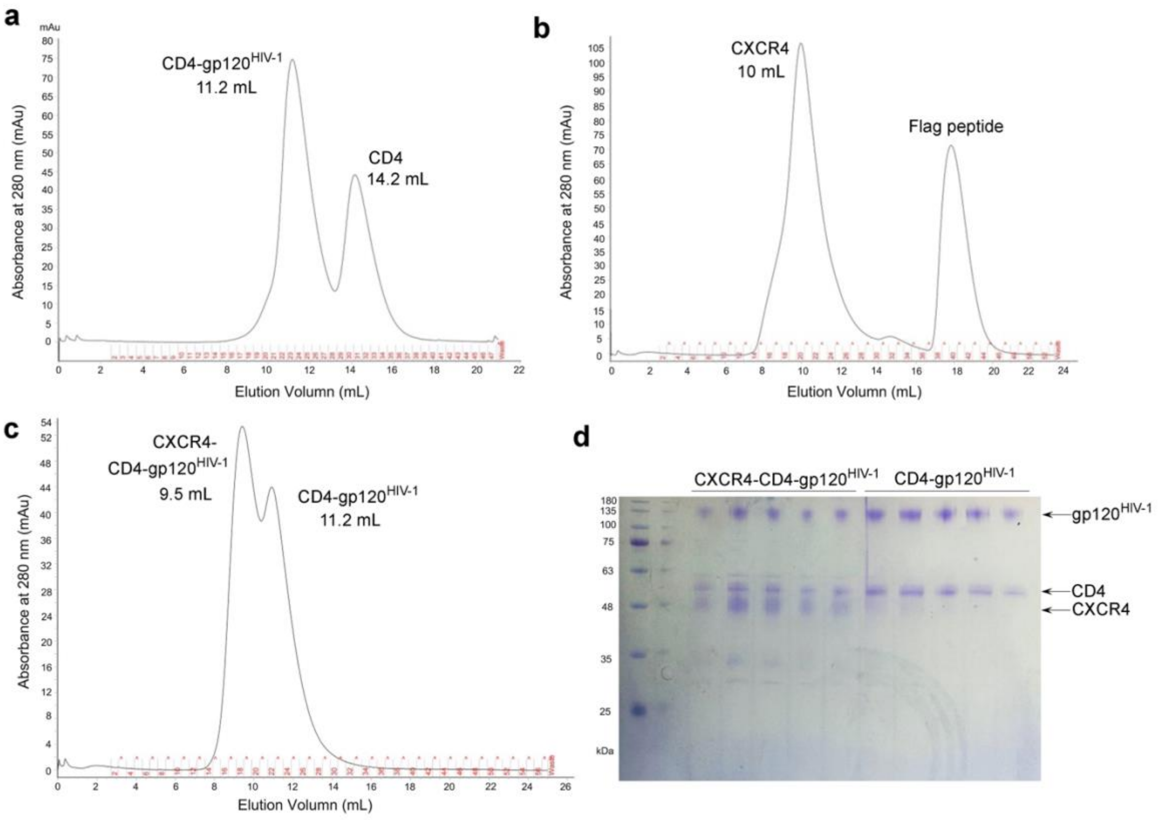

Supplementary Figure 10. **Purification of CXCR4-gp120^{HIV-1}-CD4 complex. a** Size exclusion chromatography (SEC) of gp120^{HIV-1}-CD4 complex, with the elution peak at 11.2 ml. **b** SEC of CXCR4 tetramer, with the elution peak at 10 ml. **c** SEC of the CXCR4-gp120^{HIV-1}-CD4 complex, featuring an elution peak at 9.5 ml. **d** SDS-PAGE results showing bands corresponding to CXCR4, gp120 $^{HIV-1}$, and CD4.

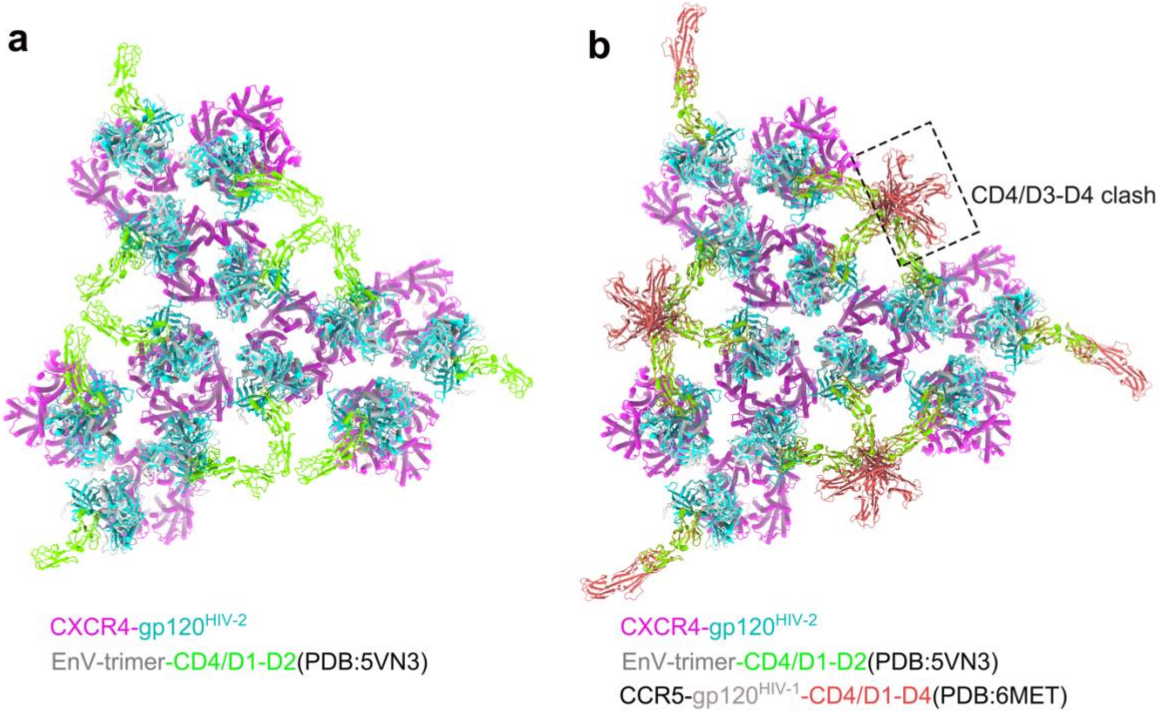

Supplementary Figure 11. **Potential high-order complex of CXCR4-gp120^{HIV-2}-CD4. a** Structural modeling of the potential high-order oligomerization of the CXCR4-gp120^{HIV-2}-CD4/D1-D2 complex. **b** Modeling of the high-order state of the CXCR4-gp120^{HIV-2}-CD4/D1-D4 complex, highlighting the clash between the CD4/D3-D4 domains.

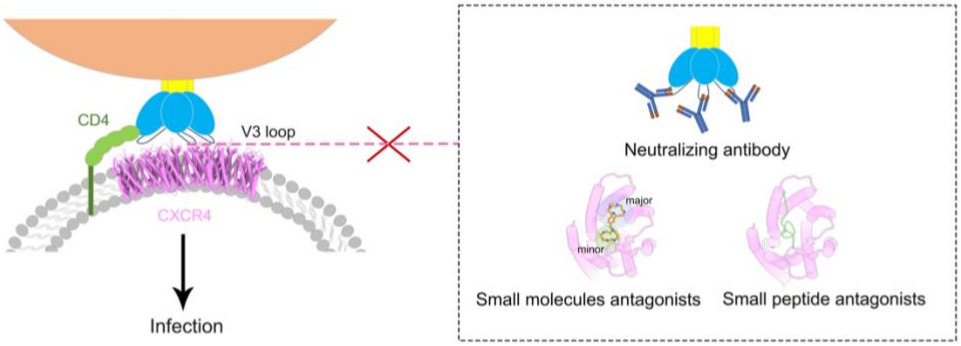

Supplementary Figure 12. Schematic of HIV-2 Env–CXCR4 recognition and inhibition strategies. The HIV-2 envelope glycoprotein (Env) interacts with the CD4 receptor and the CXCR4 co-receptor via its V3 loop to promote infection (left). Blocking this interaction can be achieved through neutralizing antibodies targeting Env, as well as small-molecule or small-peptide antagonists that bind to the CXCR4 receptor (right), preventing HIV entry.

Supplementary Table 1 | Cryo-EM data collection, refinement and validation statistics

	CXCR4 tetramer (EMDB-48180) (PDB 9MDU)	CXCL12-CXCR4 (8:4) (EMDB-48220) (PDB 9MEU)	CXCL12-CXCR4 (8:8) (EMDB-48182) (PDB 9ME1)
Data collection and processing			
Magnification	81,000	105,000	105,000
Voltage (kV)	300	300	300
Electron exposure (e–/Å^2)	59	52.8	52.8
Defocus range (μm)	-1.0 to -1.5	-0.8 to -2.2	-0.8 to -2.2
Pixel size (Å)	1.07	0.826	0.826
Symmetry imposed	C1	C4	C4
Initial particle images (no.)	2,358,042	1,980,480	1,980,480
Final particle images (no.)	734,989	135,936	181,863
Map resolution (Å)	2.87	3.37	3.46
FSC threshold	0.143	0.143	0.143
Refinement			
Initial model used (PDB code)	AF2	AF2	AF2
Model resolution (Å)	2.87	3.37	3.46
FSC threshold	0.143	0.143	0.143
Model resolution range (Å)	2.3 to 3.0	2.5 to 6.0	2.5 to 6.0
Map sharpening *B* factor (Å^2)	-116.2	-122	-173.8
Model composition			
Non-hydrogen atoms	8,570	21,155	12,027
Protein residues	1,059	2,768	1,644
Ligands	NA	NA	NA
B factors (Å^2)			
Protein	80.11	214.57	94.92
Ligand	NA	NA	NA
R.m.s. deviations			
Bond lengths (Å)	0.006	0.005	0.005
Bond angles (°)	0.962	1.037	0.906
Validation			
MolProbity score	1.83	1.52	1.51
Clashscore	6.19	3.26	3.06
Poor rotamers (%)	0	0.38	0.36
Ramachandran plot			
Favored (%)	93.86	94.04	93.75
Allowed (%)	6.14	5.92	6.18
Disallowed (%)	0	0.04	0.06

	Global-CXCR4-gp120^{HIV-2} (EMDB-48215) (PDB 9MEJ)	CXCR4-gp120^{HIV-2}/V3 loop (EMDB-48218) (PDB 9MEN)	CD4-CXCR4-gp120^{HIV-2} (EMDB-48219) (PDB 9MET)
Data collection and processing			
Magnification	81,000	81,000	81,000
Voltage (kV)	300	300	300
Electron exposure (e–/Å^2)	60.26	60.26	60.3
Defocus range (μm)	-0.8 to -2.2	-0.8 to -2.2	-0.8 to -2.2
Pixel size (Å)	0.725	0.725	0.725
Symmetry imposed	C1	C1	C1
Initial particle images (no.)	1,231,219	1,231,219	1,219,168
Final particle images (no.)	38,914	38,914	41,735
Map resolution (Å)	3.99	3.51	5.65
FSC threshold	0.143	0.143	0.143
Refinement			
Initial model used (PDB code)	AF2	AF2	AF2
Model resolution (Å)	3.99	3.51	5.65
FSC threshold	0.143	0.143	0.143
Model resolution range (Å)	3.0 to 7.0	3.0 to 7.0	3.5 to 9.0
Map sharpening *B* factor (Å^2)	-80.5	-92	-303
Model composition			
Non-hydrogen atoms	12,137	9,281	19,072
Protein residues	1,495	1,144	2,370
Ligands	NA	NA	NA
B factors (Å^2)			
Protein	341.15	157.82	1014.24
Ligand	NA	NA	NA
R.m.s. deviations			
Bond lengths (Å)	0.005	0.005	0.011
Bond angles (°)	1.258	1.086	1.288
Validation			
MolProbity score	1.74	1.67	1.68
Clashscore	6.31	4.72	6.37
Poor rotamers (%)	0.75	1.27	0
Ramachandran plot			
Favored (%)	94.13	94.97	95.33
Allowed (%)	5.87	5.03	4.59
Disallowed (%)	0	0	0.08
