# Supplementary material for: CXCR4 mediated recognition of HIV envelope spike and inhibition by CXCL12
Source: Nat Commun. 2025 Sep 30;16:8653. doi: 10.1038/s41467-025-63815-2 (PMC12485088; doi:10.1038/s41467-025-63815-2)
Supplement: Supplementary file 3 — Description of Supplementary Information [file 41467_2025_63815_MOESM3_ESM.pdf]

Description of Additional Supplementary Files

Supplementary Data 1: ChemDraw file of IT1t (.cdx format)
Supplementary Data 2: ChemDraw file of AMD3100 (.cdx format)
Supplementary Data 3: Constructs and primers used in this study
